# Transcatheter Closure of Secundum Atrial Septal Defects in Pediatric Patients: A 15-Year Single-Center Experience

**DOI:** 10.7759/cureus.57150

**Published:** 2024-03-28

**Authors:** Eser Doğan, Ertürk Levent

**Affiliations:** 1 Department of Pediatric Cardiology, Faculty of Medicine, Ege University, Izmir, TUR

**Keywords:** transcatheter closure device, asd closure, transcatheter, pediatric, atrial septal defect

## Abstract

Purpose: Transcatheter closure is the first-line treatment option for hemodynamically significant secundum atrial septal defects (ASDs). This study examines our center’s experience with this procedure over the last 15 years.

Materials and methods: Pediatric patients aged 0-18 years with secundum ASDs who were planned for transcatheter closure in our clinic between January 2007 and January 2023 were retrospectively evaluated.

Results: Transcatheter secundum ASD closure was planned for a total of 334 patients during the study period: 191 girls (57.2%) and 143 boys (42.8%). Their mean age was 8.08±3.9 years, and their mean weight was 30±15.6 kg. Defect diameter measured transesophageally ranged from 5 to 35 mm, with a mean of 12.56±4.02 mm. Transesophageal echocardiographic examination revealed a single secundum ASD in 319 patients (95.5%) and multiple secundum ASDs in 15 patients (4.5%). In 11 patients (3.3%), the procedure was terminated before initiating transcatheter ASD closure because of insufficient vena cava rims or a very large or multi-fenestrated defect. The 323 patients (96.7%) who underwent transcatheter ASD closure had a mean pulmonary artery pressure of 15.1±4.0 mmHg and a mean Qp/Qs ratio of 1.97±0.56. The procedure failed in four patients (1.3%) because of device embolization (n=2) or the inability to properly position the device (n=2). Major complications other than device embolization observed during or after transcatheter closure included anesthetic-induced respiratory depression (n=1) and total atelectasis of the lung (n=1). No new major complications were detected during the patients’ long-term follow-up.

Conclusion: With appropriate patient and device selection, transcatheter closure is a safe and effective treatment for secundum ASD and should be the first treatment of choice.

## Introduction

Atrial septal defects (ASDs) account for 6-10% of all congenital heart disorders [1]. It is twice as frequent in females as in males. Secundum defects are the most common type of ASD and are relatively easier to treat compared to the other types [2]. Secundum ASD closure can be performed surgically or percutaneously. Although there are certain criteria regarding the timing and method of secundum ASD closure, the decision is made for each patient individually by evaluating their clinical and hemodynamic status. It is generally appropriate to close secundum ASDs larger than 7-8 mm after the age of four years [3,4]. However, closure is recommended if the catheter-derived pulmonary-to-systemic flow ratio (Qp/Qs) is higher than 1.5 [5]. If this ratio is low, the defect is hemodynamically insignificant, and closure is not indicated. Very large defects can even be closed during infancy. The use of occlusion devices in recent years has further reduced the age at ASD closure to a mean age of approximately six years [6,7]. Transcatheter closure is currently the first treatment of choice. Patients who are not eligible for closure by this method, have large defects, or have rim insufficiency, are candidates for surgical closure.

This study aimed at evaluating patients with secundum ASD closed by transcatheter occlusion and to share the experience of the pediatric cardiology department of Ege University Faculty of Medicine, Izmir, Turkey, with the literature.

## Materials and methods

Patients aged 3-18 years with isolated ASD secundum who were taken to the angiography room of the Pediatric Cardiology Department of Ege University Faculty of Medicine Hospital for transcatheter closure between January 2007 and January 2023 were included in the study. Patients with any other congenital cardiac abnormality in addition to isolated ASD secundum were excluded from the study. Patients with missing data were also excluded. Written informed consent was obtained from the patients and their parents or guardians. The study was approved by the medical research ethics committee of Ege University Faculty of Medicine (decision no: 23-12T/51, decision date December 14, 2023).

Patients were evaluated by echocardiography before, during, and after the procedure. Transthoracic echocardiography (TTE) and transesophageal echocardiography (TEE) were performed using a Vivid e9 echocardiography device (GE, Vingmed Ultrasound, Norway). We can safely use our TEE probe on patients weighing over 10 kg. Measurements were made according to the standards of the American Society of Echocardiography. The decision for ASD closure was made in patients with right ventricular loading and signs of dilatation and those with a pronounced left-right shunt and Qp/Qs greater than 1.5. ASDs other than secundum ASD and patients with Eisenmenger syndrome or concomitant cardiac pathologies requiring heart surgery were not included in the study. Closure was planned for patients with rims larger than 5 mm other than aortic rims. Patients with floppy rims, multiple defects, or large defects were also evaluated by detailed TEE examination and balloon sizing for eligibility for closure. After the procedure, TEE and TTE were performed again to confirm the device position and evaluate for any residual shunt.

The patients' complaints before the procedure, age, sex, weight, height, physical examination findings, electrocardiogram (ECG) records, defect diameter, the suitability of the rims of the defect, right heart dimensions, presence of tricuspid valve insufficiency detected by echocardiography, and the brand and size of the device used were recorded. The procedures were performed in the angiography room under anesthesia as per routine protocol.

Sedation was administered to all patients. ASD diameter and the aortic and vena cava rims were measured by transesophageal echocardiography. If patients were eligible for transcatheter closure, the procedure was initiated. Under local anesthesia, an introducer sheath was placed in the right femoral vein. A multipurpose catheter was advanced to the inferior vena via the femoral venous route to access the right atrium/superior vena cava, right atrium/right ventricle, and pulmonary artery. Pulmonary artery pressure, right ventricular pressure, Qp/Qs, and pulmonary/systemic vascular resistance ratio (PVR/SVR) and index (PVRI/LVRI) values were determined, and the procedure was terminated in patients with any contraindication to transcatheter closure. In patients with Qp/Qs greater than 1.5 and no such contraindications, we proceeded with the closure procedure.

A rigid guidewire was passed through the right femoral vein to the left atrium using the multipurpose catheter. In all patients, the diameter of the defect was measured by balloon sizing using the stop-flow technique, and the appropriate device was selected accordingly. The delivery system with the device was advanced through to the end of the long sheath and then over the guidewire, and the left atrial disc was opened. The right atrial disc was then opened at the appropriate angles under TEE guidance to position the device in the interatrial septum. Before the device was released from the carrier system, TEE or TTE and fluoroscopy were used to check its position and placement in the interatrial septum and assess for the presence of residual flow through the interatrial septum, compression of the aorta or mitral and tricuspid valves, and obstruction of the superior and inferior vena cava orifices. The stability of the device in the interatrial septum was also assessed by the Minnesota maneuver. If the results of these controls were favorable, the device was disengaged from the delivery cable. During the procedure, 50 units/kg of heparin was administered intravenously. Infective endocarditis prophylaxis was administered in all patients. After the procedure, patients used aspirin at a dose of 3-5 mg/kg/day orally for six months.

Statistical analysis

Statistical analyses were performed using the IBM SPSS Statistics for Windows, Version 25 (Released 2017; IBM Corp., Armonk, New York). In descriptive statistics, continuous variables are presented as mean ± standard deviation, and categorical variables are expressed as numbers and percentages.

## Results

**Figure 1:**
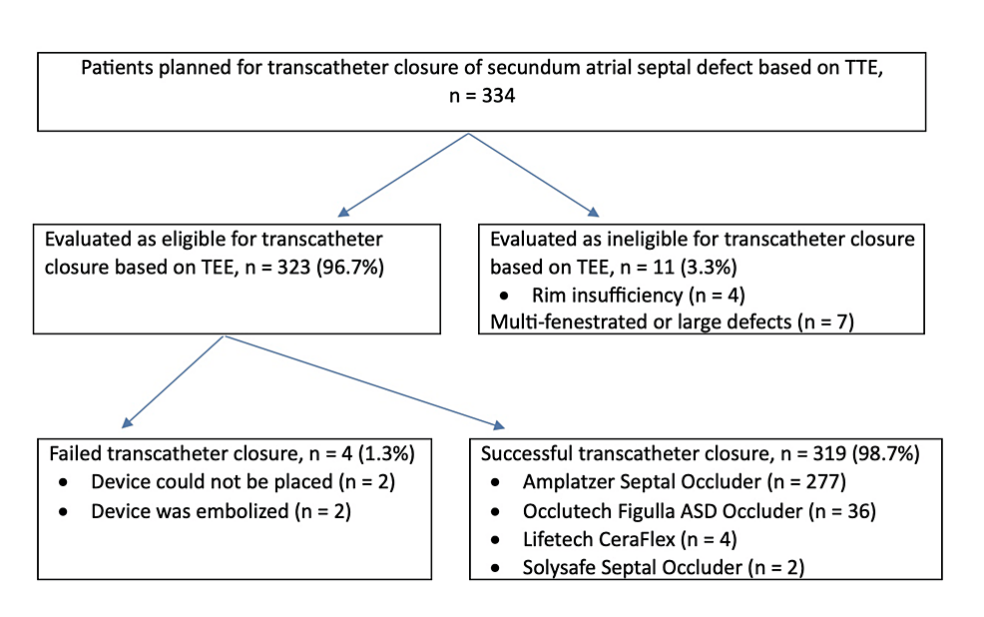
Flow chart of patient selection for transcatheter atrial septal defect closure and devices used TTE: transthoracic echocardiography, TEE: transesophageal echocardiography.

A total of 334 patients were prepared for transcatheter ASD secundum closure in the angiography room. The patient group consisted of 191 girls (57.2%) and 143 boys (42.8%). The patients' mean age at the time of the procedure was 8.08±3.9 years, and their mean weight was 30±15.6 kg. Defect diameter measured transesophageally ranged from five to 35 mm, with a mean of 12.56±4.02 mm. Among the patients in whom transesophageal measurements met the sought criteria and the transcatheter closure procedure was initiated, the mean pulmonary artery pressure measured via catheter was 15.1±4.0 mmHg, and the mean Qp/Qs was 1.97±0.56. On transesophageal echocardiography (TEE), a single secundum-type ASD was detected in 319 (95.5%) of the patients and two or more secundum ASDs were detected in 15 patients (4.5%). In total, eleven patients (3.3%) thought to be eligible for transcatheter closure based on preoperative TTE were found to not be eligible for transcatheter closure according to intraoperative TEE measurements. In our clinic, patients are evaluated by experienced pediatric cardiologists with TTE before the decision to perform angiography is made. Patients who initially have indications for surgery are discussed at the pediatric cardiac surgery council, and surgical closure is performed.

Transcatheter ASD closure was initiated in 323 patients (96.7%). Of these, the procedure was unsuccessful in only four patients (1.3%) (Figure 1). In two of those cases, the device could not be positioned in the interatrial septum despite attempting various maneuvers. In one patient, the device embolized to the left atrium during angiography. In the other patient, the occluder was placed, and its position was confirmed, but a TTE examination performed upon the sudden onset of chest pain at hour six of ward observation showed the device had embolized to the left ventricle. The two patients with device embolization underwent emergency surgery by the pediatric cardiovascular surgery department to retrieve the device and close the ASD. After surgical removal of the device, no residual defect or new valve insufficiency was detected in the patients.

Of the 323 patients who underwent ASD closure, 315 patients had no residual defect detected in examinations performed while in the angiography room. In eight patients, hemodynamically insignificant residual shunt was detected after the device was deployed, and these patients required no re-intervention during follow-up. Transcatheter closure was performed in eight of the 15 patients with multiple ASDs. In eight patients with two secundum ASDs, a single device was used to occlude both ASDs in six patients, and separate devices were used to close each ASD in the two other patients.

The devices used in the 319 patients who underwent successful ASD closure included the Amplatzer Septal Occluder (ASO; AGA Medical Corporation, Golden Valley, Minnesota) in 277 patients (86.8%), the Occlutech Figulla device (Occlutech GmbH, Jena, Germany) in 36 patients (11.3%), the CeraFlex™ ASD occluder (LifeTech Scientific Co., Shenzhen, China) in four patients (1.3%), and the Solysafe Septal Occluder (Swissimplant AG, Solothurn, Switzerland) in two patients (0.6%). Devices were selected from those that were commercially available and could be obtained at that time. Major complications were evaluated in the vascular, cardiac, respiratory, neurological, nephrological, and infectious categories. During the angiography procedure, the only major complications observed were total atelectasis of the right lung in one patient and sudden respiratory depression believed to be secondary to the anesthetic in another patient. Transient premature atrial beats or supraventricular tachycardia due to device placement or the preceding catheter manipulations were observed but did not persist in any patient. Thrombus or bleeding from the femoral vein access site was not observed. None of the patients died during or after the angiography procedure. The complications developed were not associated with the devices used. Patients had follow-up examinations at one and six months after catheterization and yearly thereafter. The patients used aspirin for six months postoperatively and none developed a thrombus. Patients were evaluated for the development of arrhythmias with an electrocardiogram (ECG) at outpatient clinic visits and Holter ECG at intervals of one to two years. No life-threatening malignant arrhythmias were detected. No new major complications were observed during long-term follow-up. The follow-up time after angiography ranged from six months to 15 years.

## Discussion

ASD is one of the most common congenital heart diseases and can be treated using a transcatheter or surgical approach [8]. Patients with ASD are generally asymptomatic in infancy and early childhood. If the amount of left-to-right shunt is not high, ASD is difficult to diagnose in the pediatric age group. With age, a large ASD with high left-to-right shunt causes arrhythmia, right heart failure, paradoxical embolism, pulmonary hypertension, and higher mortality compared to the normal population [9]. To prevent these unfavorable outcomes, defects with a Qp/Qs ratio above 1.5 tend to be closed at an earlier age. Transcatheter ASD closure was first performed in 1974 [10]. The frequency of transcatheter ASD closures is steadily rising, and the types of occlusion devices used are increasing. TEE is used during transcatheter closure to evaluate the size of the ASD, the rims, and the position of the device during deployment. All ASD closure procedures performed in our clinic were performed under TEE guidance.

Compared to surgical closure, transcatheter closure is reported to be less traumatic, does not require intensive care, provides faster recovery, and causes fewer complications [11]. Despite these advantages of transcatheter ASD closure, the rate of complications may increase for reasons such as inappropriate patient selection, wrong choice of procedure, and lack of experience [12]. Various types of complications have been described in the literature, including vascular (bleeding, vascular injury), cardiac (cardiac arrest, complete atrioventricular block, pericardial effusion, need for emergency heart surgery), respiratory (pneumothorax, respiratory failure), neurological, nephrological, infectious, and anesthetic- and device-related complications. Cardiac complications are most commonly reported [13]. Among the patients in our study, we observed respiratory complications, anesthesia-related complications, and the need for emergency surgery due to device embolization. Studies with large patient samples have reported no deaths due to transcatheter ASD closure [14]. Our findings further support this, indicating that transcatheter ASD closure is a safe and effective technique. Transcatheter ASD closure should be the first treatment of choice in eligible patients.

The ASO is the most frequently used ASD occlusion device worldwide [15]. It was also determined to be the most frequently used ASD device in our study. It ranges in size from 4 to 40 mm and is delivered through a venous sheath with a gauge up to 12 French for the largest 40 mm device. The Occlutech Figulla device is very similar to the ASO and is deployed in exactly the same way, meaning that it requires no additional training for those familiar with the ASO. This may be why it was the second most commonly used device in our clinic. The CeraFlex™ occluder is newer than the ASO and there are publications reporting it can be used safely for ASD closure [16]. However, its larger long sheath makes it difficult to use in smaller patients. Solysafe septal occluder devices led to the need for surgery in patients due to embolization and dislocation and are no longer used for this reason [17]. No complications occurred in the two patients who received this type of occluder in our clinic.

With current advances in surgical techniques, postoperative morbidity and mortality rates are also very low [18,19]. Therefore, it should be noted that surgical ASD closure is also a safe and effective method in patients not eligible for transcatheter closure.

Among the limitations of this study are the relatively small sample size and the retrospective data collection. Conducting more comprehensive investigations with a larger number of children and prospective data collection would provide consolidated evidence regarding the use of these devices in the pediatric population.

## Conclusions

This single-center study on transcatheter closure of ASD secundum in children showed a high success rate of 96.7% with minimal complications. Utilizing mainly the ASO, this method demonstrated safety and efficacy, with no mortality and rare, transient minor complications. Long-term follow-up indicated no major adverse outcomes, supporting transcatheter closure as a reliable alternative to surgery for ASD secundum.
